# Is There a Relationship between Hyperventilation Syndrome and History of Acute SARS-CoV-2 Infection? A Cross-Sectional Study

**DOI:** 10.3390/healthcare10112154

**Published:** 2022-10-28

**Authors:** Edem Allado, Mathias Poussel, Aghiles Hamroun, Anthony Moussu, Ghias Kneizeh, Oriane Hily, Margaux Temperelli, Christophe Corradi, Alexandre Koch, Eliane Albuisson, Bruno Chenuel

**Affiliations:** 1 CHRU-Nancy, Exploration Fonctionnelle Respiratoire—Centre Universitaire de Médecine du Sport et Activités Physiques Adaptées, F54000 Nancy, France; 2 DevAH, Université de Lorraine, F54000 Nancy, France; 3 Department of Public Health, Epidemiology, Health Economics and Prevention, Regional and University Hospital Center of Lille, Lille University, F59000 Lille, France; 4 CHRU-Nancy, Direction de la Recherche Clinique et de l’Innovation, F54000 Nancy, France; 5 CNRS, IECL, Université de Lorraine, F54000 Nancy, France; 6 Département du Grand Est de Recherche en Soins Primaires (DEGERESP), Université de Lorraine, F54000 Nancy, France

**Keywords:** COVID-19, hyperventilation, long COVID

## Abstract

Following COVID-19 infection, many patients suffer from long-lasting symptoms that may greatly impair their quality of life. Persisting dyspnea and other functional respiratory complaints can evoke hyperventilation syndrome (HVS) as a putative contributor to long-COVID presentation in COVID-19 survivors. We aimed to assess the possible relationship between HVS and previous acute COVID-19 infection. We designed a cross-sectional, single-center study, including all patients consecutively referred to our Lung Function and Exercise Testing Department between January and June 2021. Participants completed a systematic Nijmegen Questionnaire, a modified Medical Research Council dyspnea scale assessment, a post-COVID screening questionnaire, and performed a standardized lung function test. The population was divided according to HVS diagnosis, defined as a Nijmegen score of > 23/64. The occurrence of previous COVID-19 infection was compared according to the Nijmegen score after adjustment for potential confounders by multivariate logistic regression. In total, 2846 patients were included: 1472 men (51.7%) with a mean age of 56 (±16.6) years. A total of 455 patients (16%) declared a previous SARS-CoV-2 infection, and 590 patients presented a positive score (>23/64) in the Nijmegen Questionnaire (20.7%). Compared with COVID-19-free patients, there was an increased occurrence of HVS+ in cases of COVID-19 infection that did not require hospitalization (aOR = 1.93 [1.17–3.18]). The results of this large-scale, cross-sectional study suggest an association between HVS diagnosis and a history of COVID-19 disease in patients who were not hospitalized.

## 1. Introduction

The sudden outbreak of the COVID-19 pandemic has pressured worldwide health systems as never before to urgently handle patients in critical status [1,2]. The acute clinical presentation of COVID-19 has been rapidly characterized, encompassing a wide spectrum from asymptomatic infection to fatal disease, with a dominant respiratory feature involving worsening arterial hypoxemia that eventually leads to acute respiratory distress syndrome (ARDS) with a high mortality rate [3,4,5,6]. In contrast, chronic consequences of the acute insults resulting from SARS-CoV-2 infection have lately emerged, primarily from patients sharing experiences on social media and drawing attention to the media and medical–scientific community [7,8,9,10]. Then, the condition of long COVID or post-COVID was defined as the persistence of symptoms for at least 12 weeks after the onset of COVID-19 and has been acknowledged by the World Health Organization (WHO). The main objective was to help the development of care strategies aiming at the reduction in chronic or permanent health loss and the optimization of wellness among patients with COVID-19 [11,12,13]. 

The symptoms reported by patients following acute COVID infection are heterogeneous, ranging from the persistence of physical symptoms, such as anosmia/dysgeusia, fatigue, breathlessness, and chest pain, to psychological and cognitive symptoms, such as anxiety, depression, and poor memory and concentration, leading to significant limitations in daily living activities and quality of life [8,14,15,16,17]. Among the respiratory symptoms, dyspnea, chest pain, and cough are the most prevalent in survivors of hospital admission for COVID-19 [8,18,19]. However, the pathophysiology and natural history of long COVID need to be clarified [13,20]. Recent works have demonstrated that hyperventilation may be one of the mechanisms for persistent dyspnea in SARS-CoV-2 survivors, contributing to a substantial exercise limitation [21,22]. In such contexts, the putative role of hyperventilation syndrome (HVS) linked to long-COVID presentation has recently arisen [21,23]. This syndrome is the most common form of dysfunctional breathing, characterized by a variety of somatic symptoms induced by physiologically inappropriate hyperventilation and usually reproduced by voluntary hyperventilation [24,25,26,27,28]. The clinical diagnosis is currently based on a positive score in the Nijmegen Questionnaire (score > 23/64) and after the exclusion of other medical conditions responsible for hyperventilation [24,26,29].

Therefore, the current clinical question is whether hyperventilation syndrome is contributor to long-lasting dyspnea following COVID-19 presentation.

In order to test this hypothesis, we investigated the relationship between the occurrence of a hyperventilation syndrome and a previous history of SARS-CoV-2 infection by performing a systematic Nijmegen Questionnaire, a modified Medical Research Council dyspnea scale assessment and a post-COVID screening questionnaire with a large sample of patients observed in our Lung Function and Exercise Testing Department.

## 2. Materials and Methods

This was an observational cross-sectional, single-center study performed at the Nancy University Hospital, France, including all patients consecutively referred to our Lung Function and Exercise Testing Department between January and June 2021. The inclusion criteria were age over 18 years and the ability to perform a pulmonary function test in our Lung Function and Exercise Testing Department. Eligible patients needed to be able to read and speak French in order to correctly answer to the self-reported questionnaire. The exclusion criteria were incomplete questionnaire or an inability to obtain an interpretable lung function assessment.

Following inclusion, data were collected about age, sex, body mass index (BMI), smoking status (never smoker, current smoker, or former smoker), history of COVID-19 infection (whether previous COVID-19 presentation; if yes, date COVID-19 began), type of diagnosis assessment (PCR-confirmed COVID-19 on naso- and oropharyngeal swab or medical imaging diagnosis), and presence and level of current dyspnea assessed by the modified Medical Research Council dyspnea scale (five-point rating scale [30,31]). We collected the Nijmegen Questionnaire in order to assess functional respiratory complaints [29,32]. 

The study population was divided according to the diagnosis of hyperventilation syndrome (Nijmegen Questionnaire score > 23/64). After physical examination, questionnaire completion was verified a posteriori by the physician, without knowledge of the COVID-19 history of the patient. 

Patients performed forced spirometry according to current guidelines [33]. The presence of an obstructive ventilatory defect (OVD) at the pulmonary function test was collected. An OVD was defined by a low ratio between the first–second forced expiratory volume and the forced vital capacity (FEV1/FVC) of less than 0.7. 

Both descriptive and comparative analyses were conducted according to the nature and distribution of the variables. Qualitative variables are described with frequencies and percentages; quantitative variables with normal distribution are reported as the mean (± SD) or as the median and interquartile range (IQR), when appropriate. The chi-square test or Fisher’s exact test with, if necessary, the exact calculation of Fisher, was used for ordinal or nominal data analysis. We used the Student’s t-test to compare age and BMI; we used the Mann–Whitney test for the Nijmegen Questionnaire score and the interval between the diagnosis of COVID-19 (days). The results of univariate analyses are displayed with odds ratios (ORs) and their corresponding 95% confidence interval (95% CI). Then, a binary logistic regression analysis was performed with HVS as the outcome (HVS+ or HVS−), and the potential confounders identified by a significance level (*p* ≤ 0.05) in univariate analyses and/or clinical relevance. The Hosmer–Lemeshow goodness-of-fit test for logistic regression was performed. The Fisher’s exact test with a 5% two-sided significance level had 79.11% power to detect the difference between a group 1 proportion, π₁, of 0.19 and a group 2 proportion, π₂, of 0.22 when the sample sizes in each group was 2842.

The significance level was set to 0.05 for the entire study. IBM™ SPSS Statistics v23 (IBM, Chicago, IL, USA) was used for the data analysis. 

In order to clarify the consistency of the data obtained from consultations during the period of the study, in particular the absence of temporal bias, presenting scatter graphs of clinical characteristics as a function of the inclusion time. We defined the first wave of COVID-19 as less than 300 days and the second wave beyond 300 days (corresponding to the 10 months observed in France between these two waves). 

All data used were obtained from the medical records. No supplementary examination was necessary for patients to meet the inclusion criteria. This study was registered with the Information Technology and Freedoms Commission for the University Hospital of Nancy (2021PI224-203) and registered with Clinicaltrials.gov (NCT05224830). The protocol of this study was designed in accordance with the general ethical principles outlined in the Declaration of Helsinki. The protocol of this study was approved by the Information Technology and Freedoms Commission. All patients gave their consent for the use of their medical data during the period they received medical care at the University Hospital.

## 3. Results

During the 20-week study period, 2917 patients were referred to our Pulmonary Function Testing and Exercise Physiology Department. Seventy-one patients were excluded from the analysis due to incomplete questionnaires or noninterpretable lung function assessments. All inclusions were systematic and consistent over time. A total of 2846 patients were included: 1472 men (51.7%) with a mean age of 56 (±16.6) years and an average BMI of 28.2 (±7.1) kg/m^2^.

The baseline demographic and clinical characteristics of the patients are presented in Table 1. A total of 456 patients (16%) declared a previous COVID-19 infection, with a range between the diagnosis and the visit to the Pulmonary Function and Exercise Testing Department of 15–489 days and the existence of two waves. In this sample, 451 (98.9%) patients had laboratory-confirmed COVID-19 and 5 patients had medical imaging compatible with COVID-19 lung damage. Among them, 203 patients had been hospitalized during the acute phase (45.1%).

Overall, 590 patients presented a positive score (>23/64) on the Nijmegen Questionnaire (20.7%), with no significant difference in the prevalence of a previous acute SARS-CoV-2 infection between the two HVS statuses: 17.1% for the HVS+ group and 15.7% for the HVS− status. The HVS+ status presented a greater ratio of women, lower age, a higher BMI, a more pronounced dyspnea, and a greater proportion of patients with a history of COVID-19 infection who did not require hospitalization (Table 1). As shown in Table 2, mild COVID-19 injury was more prevalent in patients with HVS than in patients after severe COVID-19 disease requiring hospitalization. After adjustment, as shown in Table 2, HVS+ was significantly associated with a history of previous COVID-19 infection, in opposition to its clinical management (Hosmer–Lemeshow = 0.516, Table 3). Compared with COVID-19-free patients, there was an increased frequency of HVS+ in cases of COVID-19 infection that did not require hospitalization (aOR = 1.93 [1.17–3.18], S) and a decreased occurrence of HVS+ in cases of COVID-19 infection that required hospitalization (aOR = 0.84 [0.497–1.41], NS).

## 4. Discussion

This work is, to the best of our knowledge, the first to investigate a possible relationship between HVS and a history of acute SARS-CoV-2 infection. While we found a similar rate of previous acute SARS-CoV-2 infection in both statuses of patients, irrespective of HVS diagnosis (16.9 and 15.7%, respectively, in the HVS+ and HVS− statuses), we observed a significant relationship between previous COVID-19 and HVS+, but only with patients who did not require hospitalization.

In our sample, the prevalence of HVS diagnosis (20.7%) was twice as high as the reported prevalence in the general adult population, ranging from 6% to 10% [26,34]. However, our large sample is not representative of the general population, because it included patients with known and unknown respiratory diseases suffering from dyspnea. If some patients were finally classified as healthy subjects, because they did not present any lung function limitation, they may not represent the healthy general population. Moreover, because the patients included in this study were referred to our clinical department to perform a pulmonary function test, a large number of patients with obstructive or restrictive respiratory diseases were studied. Concomitantly, overestimation of patients with dyspnea and underestimation of asymptomatic patients following SARS-CoV-2 infection were suspected, compared with the general population. 

Secondly, if the Nijmegen score is used as a screening tool to help the clinical diagnosis of a hyperventilation syndrome with a sensitivity of 91% and specificity of 95% in the original publication [29], a positive Nijmegen Questionnaire score (>23/64) is demonstrated in patients with panic disorder [35,36] and in many asthmatics, especially with poor asthma control [34,37,38]. The prevalence of a positive score on the Nijmegen Questionnaire in our work (20.7%) is close to the prevalence of hyperventilation in asthmatics patients, ranging from 20% to 34% [34,38]. In COPD patients, dysfunctional breathing was more frequently found than in asthma patients and healthy people, with detection by an isolated positive Nijmegen Questionnaire score reaching 50% of patients [39]. Accordingly, several case reports have highlighted such an association between HVS and COPD [40].

On the other hand, it has been widely accepted that the sum score of the Nijmegen Questionnaire is a relevant tool to represent a subjective score of “functional respiratory complaints” associated with stress, anxiety, and respiration [32]. Because the prevalence of anxiety and depression among patients with chronic obstructive pulmonary disease is significantly higher than among the general population [41,42,43], we suspect that the prevalence of a positive Nijmegen Questionnaire score could have been overestimated in such a sample of patients (those referred to our Lung Function Department). 

In the same manner, if the Nijmegen Questionnaire is accepted as exploring the psychic dimension of breathing and its response to stress, the high rate of positive scores in patients with a history of SARS-CoV-2 infection could be related to the high prevalence of anxiety, insomnia, depression, and post-traumatic stress disorder that was already demonstrated among COVID-19 survivors (42%, 40%, 31%, and 28%, respectively) [44]. 

The potential link between COVID-19 and HVS has already been suspected in recent works that have clearly demonstrated the role of hyperventilation as one of the mechanisms for persistent dyspnea in COVID-19 survivors [23,45,46]. 

The specific involvement of the respiratory centers anatomically localized in the brainstem for the instability of breathing control in COVID-19 has been hypothesized. The suspected mechanisms involve inflammatory or microangiopathic alteration of the pre-Bötzinger, leading to dysregulation of the ventilatory drive [3,47]. However, further studies are needed to confirm this hypothesis. The pathophysiology of hyperventilation syndrome has not been totally clarified, but the complex interaction between respiratory, psychiatric, and physiological disturbances to the control of breathing has been commonly emphasized [27]. As a form of dysfunctional breathing, hyperventilation involved in the HVS may initially be triggered by somatic diseases, such as asthma, pneumonia, or pain, but the multifactorial etiology of the problem should not rule out the benefit of nonspecific therapies, such as exercise or regular adapted physical activity, in addition to techniques addressing voluntary breathing control [24]. 

We observed positive relationships between HVS and female sex, middle age, a high level of dyspnea, and the absence of fixed baseline obstructive ventilatory limitation, fully in agreement with current knowledge on HVS [24,25,26,27,28]. The potential association between a previous SARS-CoV-2 infection and HVS is a more novel finding; this association is highlighted in the nonhospitalized population. Matta et al. recently demonstrated that persistent clinical symptoms following SARS-CoV-2 infection may be more associated with the belief in having been infected than having laboratory-confirmed COVID-19 disease [48]. Whether nonspecific mechanisms, less severe COVID-19 disease, and/or the lack of institutional care management is involved, better knowledge of the pathophysiology of persistent clinical symptoms after COVID-19 is required. A very recent study also found that shortness of breath was more common among nonhospitalized patients aged 20 years or older, with a positive, as opposed to negative, test result for SARS-CoV-2 from 31 to 150 days after testing [49].

In addition to the large sample of patients, their consecutive and systematic inclusion is the main strength of this study. Moreover, we could add to them the use of standardized pulmonary function tests, an HVS questionnaire, and systematic dyspnea assessment with a validated scale. 

However, this study had some limitations. The first limitation of our work is the lack of international guidelines for the diagnosis of HVS. In this context, we used the Nijmegen Questionnaire as the only criterion for HVS. It was introduced over 35 years ago, primarily to screen patients with hyperventilation-related symptoms in order to benefit from breathing exercises guided by capnographic feedback [50]. It was secondarily used as a screening tool to help with the clinical diagnosis of hyperventilation syndrome with a sensitivity of 91% and specificity of 95% in the original publication [29,32]. However, it has been recommended that the Nijmegen Questionnaire no longer be used as the unique criterion to diagnose HVS, and a multidimensional approach is highly recommended [29,32]. Some recent studies have emphasized an interest in cardiopulmonary exercise testing for the identification of patients with hyperventilation syndrome, demonstrating clearly inappropriate hyperventilation and eliminating other underlying organic disease [44,51]. However, if the Nijmegen Questionnaire is accepted as representing a subjective score of “functional respiratory complaints”, i.e., exploring the psychic dimension of breathing and its response to stress, it may be a pertinent tool for the follow-up with COVID-19 survivors, because a high prevalence of anxiety, insomnia, depression, and post-traumatic stress disorder has already been demonstrated among them (42%, 40%, 31%, and 28%, respectively) [44]. Therefore, as imperfect as it is, it seems to us that the Nijmegen Questionnaire is an interesting tool in the detection, even if rough, of possible HVS on a large sample of subjects.

The absence of an objective confirmation of HVS in patients with positive score on the Nijmegen Questionnaire is a second limitation. In particular, the use of cardiopulmonary exercise testing (CPET) has already demonstrated its role in the diagnosis of chronic unexplained dyspnea [52]. Recent articles and reviews have highlighted the great value of the implementation of CPET into current clinical practice to improve the diagnosis and management of dysfunctional breathing [51,53]. The characteristic pattern of exercise-induced inappropriate hyperventilation is documented by an increase in ventilation related to CO2 output (V’E/V’CO2 slope) or ventilatory equivalent for CO2 at different time points of the ramp-incremental exercise test [54]. The inclusion of CPET data in the HVS assessment should, therefore, be strongly considered in future investigations.

In addition, our work was not designed to ascertain the temporal profile of HVS and compare it with the chronology of SARS-CoV-2 infection: some patients might have had HVS before being infected by SARS-CoV-2. Secondly, the systematic inclusion of patients referred to a lung function department introduced selection bias, overestimating patients with respiratory complaints and concomitantly underestimating asymptomatic patients following COVID-19. However, because dyspnea is the cornerstone symptom of HVS and one of the most prevalent signs of long-COVID presentation [8,18,19], our systematic approach should have maximized the possibility of observing a potential relationship. Thirdly, we were not able to verify the serological status of declared previous SARS-CoV-2 infection, so we could not exclude wrong declarations of COVID-19 infection or undeclared asymptomatic episodes. Furthermore, this study did not take into consideration the type of viral variants of SARS-CoV-2 with different impacts on respiratory function. As has been recently pointed out, without appropriate controls, often associated with systematic selection bias of cases, it appears to be very difficult to clarify the mechanisms involved in post-COVID presentation, and, finally, to treat it accordingly [20,48,55]. 

## 5. Conclusions

In this large-scale, cross-sectional study, HVS seemed to be associated with a history of acute SARS-CoV2 infection only in patients who did not require hospitalization. Nonhospitalized COVID-19 survivors seemed more likely to suffer from HVS, despite having presented, a priori, a less serious form of the disease. Further studies are needed to better characterize post-COVID syndrome and its pathophysiology in order to guide clinical recommendations for optimized care.

## Figures and Tables

**Table 1:** Baseline demographic and clinical characteristics of included patients, according to HVS diagnosis (*n* = 2846).

	Total (*n* = 2846)	HVS− (*n* = 2256)	HVS+ (*n* = 590)	*p*-Value
Women	1374 (48.3)	1000 (44.3)	374 (63.4)	<0.001
Age, years	56.0 (16.6)	56.6 (16.8)	53.8 (15.8)	<0.001
Body mass index, kg/m^2^	28.2 (7.1)	28.0 (7.0)	28.9 (7.5)	0.010
Nijmegen Questionnaire score	14.5 (10.8)	10.2 (6.5)	31.3 (6.6)	<0.001
Dyspnea (mMRC score > 0)	1946 (68.4)	1400 (62.1)	546 (92.5)	<0.001
FEV1/FVC ratio < 0.7	295 (10.4)	230 (10.2)	65 (11.0)	0.569
Dyspnea (mMRC scale) Grade 0 Grade 1 Grade 2 Grade 3 Grade 4	900 (31.6) 944 (33.2) 189 (6.6) 327 (11.5) 486 (17.1)	856 (37.9) 785 (34.8) 132 (5.9) 232 (10.3) 251 (11.1)	44 (7.5) 159 (26.9) 57 (9.7) 95 (16.1) 235 (39.8)	<0.001
Smoking status Never smoker Current smoker Former smoker	1159 (40.7) 512 (18.0) 1174 (41.3)	929 (41.2) 386 (17.1) 940 (41.7)	230 (39.0) 126 (21.4) 234 (39.7)	0.058
Previous history of SARS-CoV-2 infection No COVID-19 infection COVID-19 without hospitalization COVID-19 with hospitalization	2390 (84.0) 253 (8.9) 203 (7.1)	1901 (84.3) 184 (8.2) 171 (7.6)	489 (82.9) 69 (11.7) 32 (5.4)	0.008
Interval between the diagnosis of COVID-19 (days)	221 (138)	220 (140)	227 (133)	0.613

Data are presented as *n* (%) for dichotomous variables, mean (SD) for continuous demographic variables with normal distribution and median [interquartile range] with non-normal distribution. FEV1 = forced expiratory volume in one second; FVC = forced vital capacity mMRC scale = modified Medical Research Council dyspnea scale assessment; HVS+, defined as a positive score > 23/64 on the Nijmegen Questionnaire; HVS−, defined as a negative score ≤ 23/64 in the Nijmegen Questionnaire.

**Table 2:** Univariate and multivariate analyses (*n* = 2846).

	OR [95% CI]	
	Univariate	Multivariate
Women	2.18 [1.80–2.62]	1.98 [1.61–2.44]
Age, years	0.99 [0.99–0.99]	0.98 [0.97–0.98]
Body mass index, kg/m^2^	1.02 [1.01–1.03]	0.98 [0.97–0.99]
FEV1/FVC ratio < 0.7	1.09 [0.81–1.46]	1.48 [1.06–2.06]
Dyspnea (mMRC scale) Grade 0 Grade 1 Grade 2 Grade 3 Grade 4	1.0 3.97 [2.78–5.58] 8.45 [5.44–12.97] 7.97 [5.42–11.72] 18.21 [12.82–25.88]	1.0 3.96 [2.70–5.52] 10.57 [6.69–16.69] 10.80 [7.19–16.23] 26.58 [18.16–38.91]
Previous history of SARS-CoV-2 infection No COVID-19 infection COVID-19 without hospitalization COVID-19 with hospitalization	1.0 1.46 [1.10–1.96] 0.73 [0.49–1.08]	1.0 1.93 [1.17–3.18] 0.84 [0.497–1.41]
Group interval between diagnosis of COVID-19 and inclusion	1.27 [0.78–1.96]	0.60 [0.35–1.02]

FEV1 = forced expiratory volume in one second; FVC = forced vital capacity, mMRC scale = modified Medical Research Council dyspnea scale assessment.

**Table 3:** Baseline demographic and clinical characteristics of included patients, according to HVS diagnosis, COVID-19 diagnosis, and strategy care (*n* = 2846).

	HVS−			HVS+		
	No COVID-19 Infection	COVID-19 without Hospitalization	COVID-19 with Hospitalization	No COVID-19 Infection	COVID-19 without Hospitalization	COVID-19 with Hospitalization
Number	(*n* = 1901)	(*n* = 184)	(*n* = 171)	(*n* = 489)	(*n* = 69)	(*n* = 32)
Expected number (global khi^2^)	1894	201	161	496	52	42
Women (%)	836 (44.0)	93 (50.5)	71 (41.5)	308 (63.0)	49 (71.0)	17 (53.1)
Age, years	56.7 (16.9)	50.5 (16.8)	62.84 (12.9)	54.1 (16.0)	49.6 (14.2)	57.2 (14.1)
Body Mass Index, kg/m^2^	27.8 (7.0)	27.8 (6.9)	30.61 (6.5)	28.8 (7.7)	28.9 (6.6)	29.8 (6.0)
Time interval between diagnosis of COVID-19 and inclusion (days)		196.6 (133.4)	244.2 (143.1)		217.6 (135.2)	248.7 (126.8)
FEV1/FVC ratio	91.9 (17.2)	95.1 (15.0)	101.9 (12.0)	91.4 (18.6)	95.9 (14.5)	102.0 (14.5)
Nijmegen Questionnaire score	10.1 (6.5)	11.0 (7.0)	10.0 (6.5)	31.3 (6.6)	31.8 (6.6)	30.1 (6.9)

Data are presented as *n* (%) for dichotomous variables, mean (SD) for continuous demographic variables with normal distribution and median [interquartile range] with non-normal distribution. FEV1 = forced expiratory volume in one second; FVC = forced vital capacity mMRC scale = modified Medical Research Council.

## Data Availability

Not applicable.
